# *Erwinia teleogrylli* sp. nov., a Bacterial Isolate Associated with a Chinese Cricket

**DOI:** 10.1371/journal.pone.0146596

**Published:** 2016-01-22

**Authors:** Bo Liu, Jin Luo, Wei Li, Xiu-Feng Long, Yu-Qin Zhang, Zhi-Gang Zeng, Yong-Qiang Tian

**Affiliations:** 1 Key laboratory of Leather Chemistry and engineering, College of Light Industry, Textile & Food Engineering, Sichuan University, Chengdu, Sichuan, P. R. China; 2 Department of Pharmaceutical and Biological Engineering, College of Chemical Engineering, Sichuan University, Chengdu, Sichuan, P. R. China; 3 Institute of Medicinal Biotechnology, Chinese Academy of Medical Sciences & Peking Union Medical College, Beijing, P. R. China; 4 Sichuan Industrial Institute of Antibiotics, Chengdu University, Chengdu, Sichuan, P. R. China; University of Porto, PORTUGAL

## Abstract

A bacterial isolate (SCU-B244^T^) was obtained in China from crickets (*Teleogryllus occipitalis*) living in cropland deserted for approximately 10 years. The isolated bacteria were Gram-negative, facultatively anaerobic, oxidase-negative rods. A preliminary analysis of the 16S rRNA gene sequence indicated that the strain belongs to either the genus *Erwinia* or *Pantoea*. Analysis of multilocus sequence typing based on concatenated partial *atpD*, *gyrB* and *infB* gene sequences and physiological and biochemical characteristics indicated that the strain belonged to the genus *Erwinia*, as member of a new species as it was distinct from other known *Erwinia* species. Further analysis of the 16S rRNA gene showed SCU-B244^T^ to have 94.71% identity to the closest species of that genus, *Erwinia oleae* (DSM 23398^T^), which is below the threshold of 97% used to discriminate bacterial species. DNA-DNA hybridization results (5.78±2.52%) between SCU-B244^T^ and *Erwinia oleae* (DSM 23398^T^) confirmed that SCU-B244^T^ and *Erwinia oleae* (DSM 23398^T^) represent different species combined with average nucleotide identity values which range from 72.42% to 74.41. The DNA G+C content of SCU-B244^T^ was 55.32 mol%, which also differs from that of *Erwinia oleae* (54.7 to 54.9 mol%). The polyphasic taxonomic approach used here confirmed that the strain belongs to the *Erwinia* group and represents a novel species. The name *Erwinia teleogrylli* sp. nov. is proposed for this novel taxon, for which the type strain is SCU-B244^T^ (= CGMCC 1.12772^T^ = DSM 28222^T^ = KCTC 42022^T^).

## Introduction

Resistance to pesticides in insects is a serious concern worldwide [1] and often occurs approximately 10 years after the introduction of a new pesticide [2]. The resistance mechanisms have been attributed to evolutionary changes in insect genomes, such as the alteration of drug target sites, up-regulation of degrading enzymes and the enhancement of drug excretion. Kikuchi *et al*. have shown that infection with an insecticide-degrading bacterial symbiont immediately establishes insecticide resistance in insects [1], indicating bacteria play an important role in pesticide resistance.

In a recent study, we focused on the culturable strains associated with crickets (*Teleogryllus occipitalis*), a common pest living in a deserted cropland in China. Our aim was to explore the relationship between pesticide resistance and symbionts. Among 274 isolates cultured from *Teleogryllus occipitalis*, 27 strains of genera *Lysinibacillus*, *Pseudomonas*, *Sphingobacterium*, *Exiguobacterium* and *Staphylococcus* could evidently degrade chlorpyrifos, a common insecticide used in this field for many years. One isolate (SCU-B244^T^) that could degrade chlorpyrifos was cultured on TSA (tryptone soy agar) medium in August 2012 and could not be identified to the species level. A polyphasic taxonomic approach was used to investigate the strain, with the results suggesting that SCU-B244^T^ represents a novel species of the genus *Erwinia*.

## Materials and Methods

### Isolation procedures, culture conditions and initial microbiological characterization

Three crickets (collected near 30°33’N, 103°58’E; altitude 495 m) were added to 100 mL sterile 0.85% (w/v) NaCl solution in a 250 mL flask and shaken at 220 rpm for 30 min. The supernatant which contained bacteria was plated onto TSA plates (Tryptone 1.5%, Soy Peptone 0.5%, NaCl 0.5%, Agar 1.5%, w/v, pH 7.2) and subsequently incubated at 37°C for 5 days. Glycerol stock at -80°C was adopted for long term preservation of the isolates. Exponential phase cells cultured in TSB (Tryptone 1.5%, Soy Peptone 0.5%, NaCl 0.5%, pH 7.2) medium with shaking (200 rpm, 16 h) at 37°C were harvested for DNA G+C content and ANI analysis. Phase contrast and transmission electron microscopy were used to examine cellular morphology and motility after growth on TSA medium at 37°C for 24 h. Gram staining was performed as described by Gerhardt *et al*. [3]. Oxidase and catalase activity were tested according to methods described by Smibert & Krieg [3]. TSB medium with different NaCl concentration and pH were used to test tolerance to NaCl and pH range, and the growth was determined by OD 600. To confirm the results, each experiments mentioned above were performed three times. *Erwinia oleae* (DSM 23398^T^) was used as reference strain in this study.

### 16S rRNA gene sequence analysis

DNA extraction from strain SCU-B244^T^, PCR amplification, primers used and DNA sequencing conditions of 16S rRNA gene were performed as previously described [4]. The strain was analysed using the EzTaxon server [5] (www.ezbiocloud.net/eztaxon) by comparison with 16S rRNA gene sequence data. A neighbour-joining phylogenetic tree was constructed using the method of Saitou and Nei [6] with MEGA 5.2 software [7]. Similarities were calculated using the Kimura 2-parameter [8, 9] in MEGA 5.2. Maximum-likelihood phylogenetic trees were also constructed using Kimura 2-parameter [8, 9] model and the method of Felsenstein [10] with MEGA 5.2 software. Robustness of the phylogenetic trees was evaluated by using the bootstrap resampling method of Felsenstein [11], with 1000 replicates.

The results of 16S rRNA gene sequence alignment on the EzTaxon server revealed that strain SCU-B244^T^ belongs to the family *Enterobacteriaceae*. Related 16S rRNA gene sequences for initial analysis were taken from the top 66 hits on the EzTaxon server (on May 2014, each representing a different species), with the highest similarity scores being 96.1% to 94.1%. Neighbour-joining and maximum-likelihood phylogenetic trees were constructed using the methods described above.

To refine the taxonomic position of SCU-B244^T^, other neighbour-joining and maximum-likelihood phylogenetic trees based on 16S rRNA gene sequences were constructed, including the strain SCU-B244^T^ and all culturable type strains of the genera *Erwinia* (Candidatus *Erwinia dacicola*) and *Pantoea*, based on the same methods described above.

### Multilocus sequence analysis

Multilocus sequence analysis (MLSA) of concatenated partial *atpD*, *gyrB*, *infB* and *rpoB* gene sequences enables the differentiation of the phylogenetically related genera *Erwinia*, *Pantoea* and *Tatumella*. A good congruence has previously been observed between DNA-DNA hybridization and MLSA [12–16]. In the present study, to confirm the results observed with 16S rRNA gene sequencing data, three housekeeping genes, *atpD*, *gyrB*, and *infB*, were amplified and sequenced with the primers described by Brady *et al*. [12]. Neighbour-joining and maximum-likelihood phylogenetic trees based on concatenated partial *atpD*, *gyrB* and *infB* gene sequences were constructed using the same method as described above. Strains from genera *Erwinia*, *Pantoea* and *Tatumella* were used in MLSA analysis, including SCU-B244^T^. The partial sequence of *atpD*, *gyrB* and *infB* gene of related strains were obtained from GenBank and the accession numbers are indicated on the figures.

### DNA-DNA hybridization

DNA-DNA hybridization between SCU-B244^T^ and *Erwinia oleae* DSM 23398^T^ was conducted as described by De Ley *et al*. [17] with hybridization temperature set at 67°C. Genomic DNA (OD_260/280_ = 1.8 to 1.9) was extracted using the Omega Bacterial DNA kit (E.Z.N.A.^®^)

### DNA G+C content

The DNA G+C mol% content of strain SCU-B244^T^ was determined by whole genome sequencing data.

### Physiological and biochemical analysis

API 20E, API 50CHE acid production tests and API ZYM enzymatic characteristics test (BioMérieux) were performed according to the manufacturer’s instructions. BIOLOG GN2 carbon-source utilization analysis was conducted according to the instruction manual. Cellular fatty acids analysis was also performed. Biomass for fatty acid analysis was prepared by scraping growth from TSA plates after 24h incubation at 37°C. Lipids were extracted using the method of Folch *et al*.[18]. Total lipids were converted to fatty acid methyl esters (FAMEs) with 4 mol/L HCl in 55% (V/V) methanol at 80±1°C for 10 mins. FAMEs were analyzed by GC-MS (Trace DSQII, Thermo Fisher), and detailed conditions of the fatty acids analysis by GC-MS have been previously described [19], The compounds were identified using NIST 05 database (NIST Mass Spectral Database, PC-Version 5.0, 2005, National Institute of Standardisation and Technology, Gaithersburg, MD, USA). To confirm the results, each experiment mentioned above was performed three times.

## Genome Sequencing and Average Nucleotide Identity

For the genome sequencing of SCU-B244^T^, Next Generation Sequencing (Illumina Miseq) was conducted at Majorbio Inc. and the data was used to calculate the Average Nucleotide Identity (ANI). DNA extraction method and purity were described above. ANI is a similarity measure between two genome sequences and it correlates well with DNA-DNA hybridization values. [20] A value of 70% DNA-DNA hybridization corresponds to about 95–96% ANI. [21] Genome data were taken from NCBI Genome and EzGenome web site. (www.ezbiocloud.net/ezgenome/browse_db). We used Orthologous Average Nucleotide Identity Tool (OAT software, Lee *et al*. 2015, Manuscript submitted, www.ezbiocloud.net/sw/oat) to calculate orthologous ANI values. This Whole Genome Shotgun project has been deposited at DDBJ/EMBL/GenBank under the accession LLXO00000000. The version described in this paper is version LLXO01000000.

## Results and Discussion

In total 274 isolates belonging to 29 genera were cultured from *Teleogryllus occipitalis* and a bacterial isolate (SCU-B244^T^) is herein described. Initial microbiological characterization of the strain revealed that the cells were Gram-negative, oxidase-negative, rod-shaped, catalase-positive and facultatively anaerobic, suggesting that the strain belongs to the family *Enterobacteriaceae* [22].

Neighbour-joining (S1 Fig) and maximum-likelihood (S2 Fig) phylogenetic trees of the first 66 hit with similarity values ranging from 96.1% to 94.1% revealed that strain SCU-B244^T^ and strains of the genera *Erwinia* and *Pantoea* cluster together. The diagram shows the phylogenetic relationship between SCU-B244^T^ and genera *Erwinia* and *Pantoea*.

**Fig 1:**
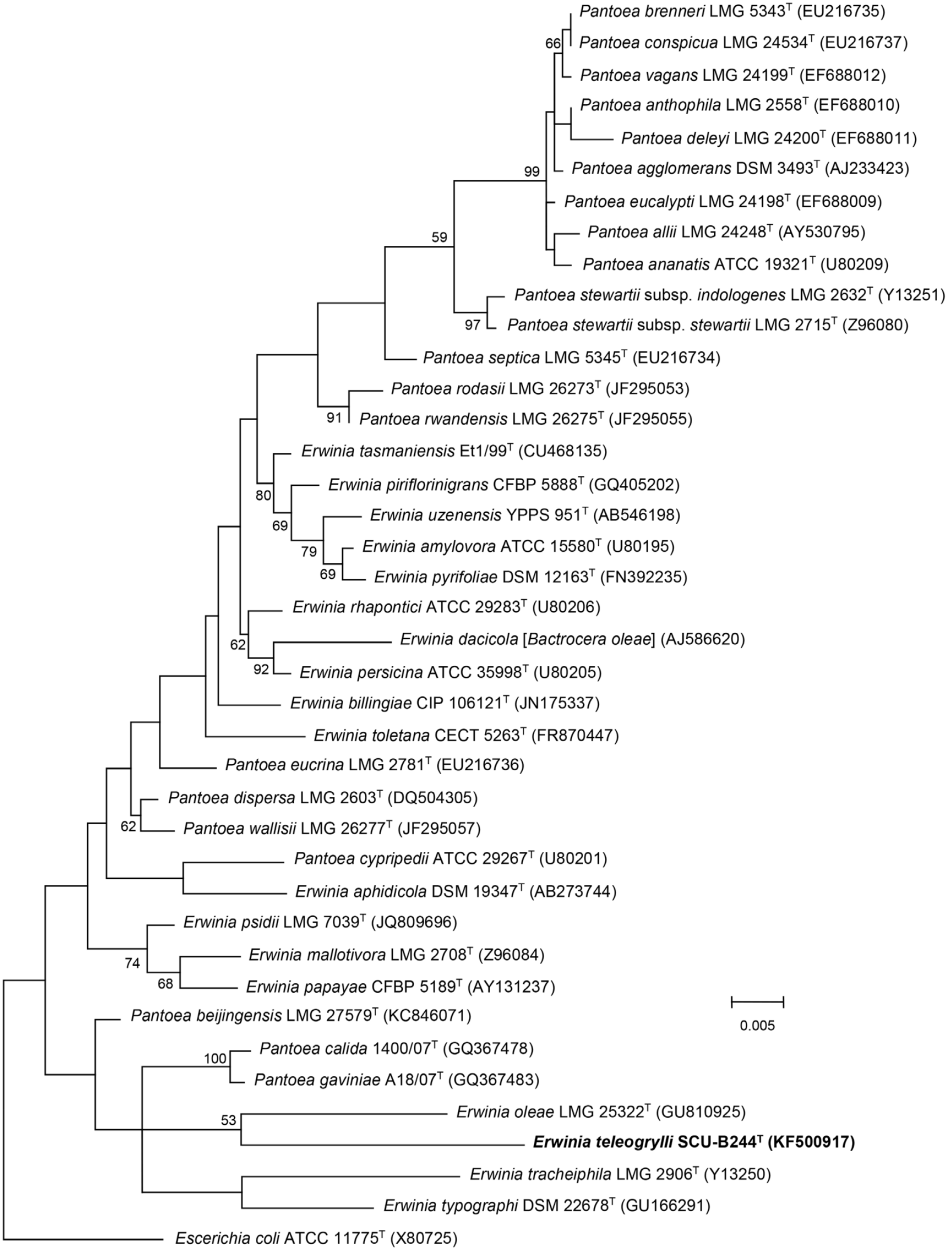
Maximum-likelihood tree based on partial 16S rRNA gene sequences of the genera *Erwinia* and *Pantoea*. The diagram shows the phylogenetic relationship between *Erwinia teleogrylli* sp. nov. and related type species of the genera *Erwinia* and *Pantoea* except *Candidatus* Erwinia dacicola. *Escherichia coli* ATCC 11775^T^ was used as the outgroup. Bar, 0.5% nucleotide substitutions. Numbers at branching points are bootstrap percentage values based on 1000 replications. Only values >50% are shown.

Further neighbour-joining (S3 Fig) and maximum-likelihood (Fig 1) phylogenetic trees, based on 16S rRNA gene sequences from members of the genera *Erwinia* and *Pantoea*, indicated that strain SCU-B244^T^ belongs to the genus *Erwinia* and is most closely related to *Erwinia oleae* (DSM 23398^T^), which is consistent with the results of the initial phenotypic analysis.

**Fig 2:**
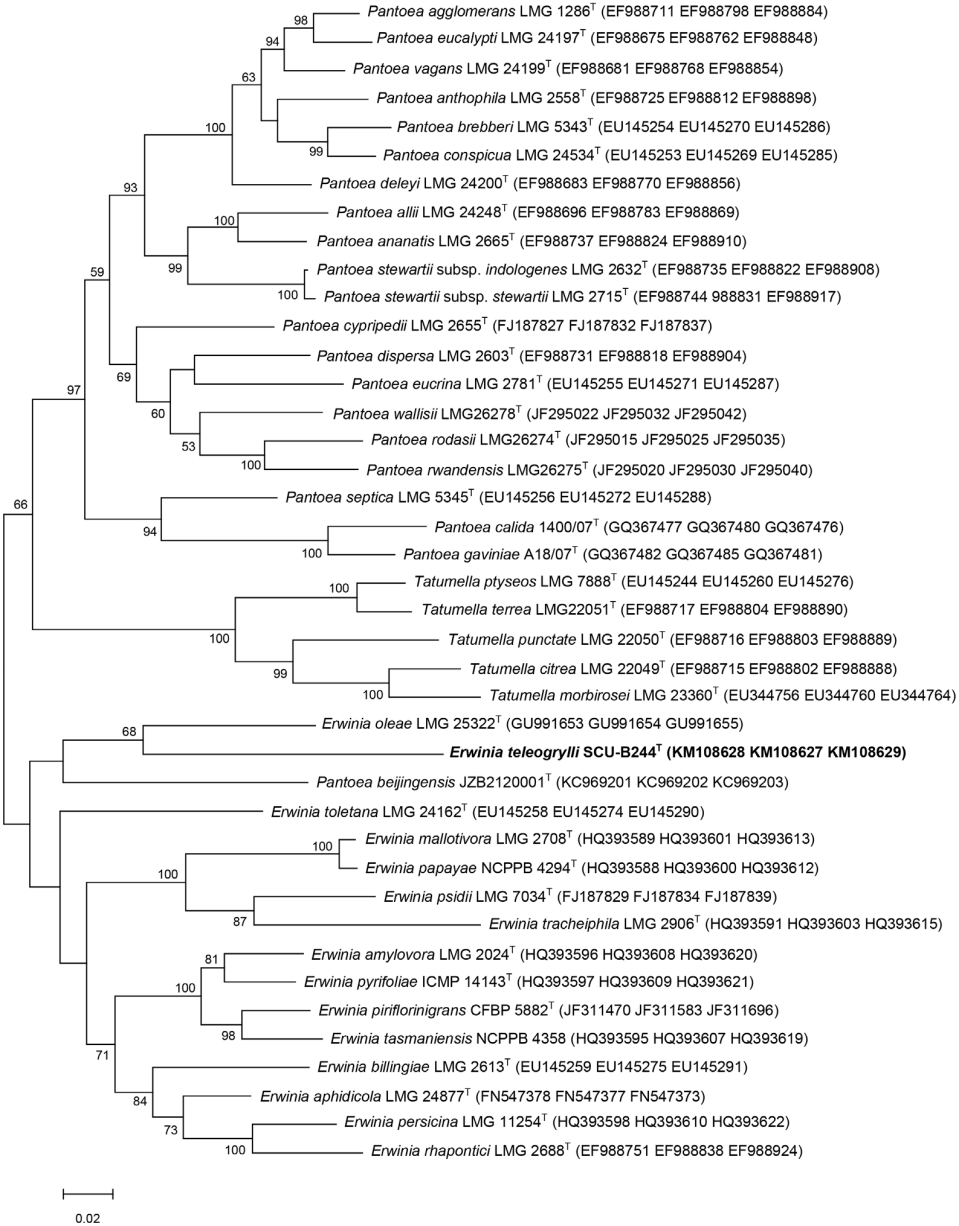
Maximum-likelihood tree based on concatenated partial *atpD*, *gyrB* and *infB* sequences from the genera *Erwinia*, *Pantoea* and *Tatumella*. The diagram shows the phylogenetic relationship between *Erwinia teleogrylli* sp. nov. and related species of the genera *Erwinia*, *Pantoea* and *Tatumella*. Bar, 0.5% nucleotide substitutions. Numbers at branching points are bootstrap percentage values based on 1000 replications. Only values >50% are shown.

In neighbour-joining (S4 Fig) and maximum-likelihood (Fig 2) phylogenetic trees based on concatenated partial *atpD*, *gyrB* and *infB* gene sequences, strain SCU-B244^T^ and *Erwinia oleae* (DSM 23398^T^) cluster together on a single branch, consistent with the previous results, suggesting strain SCU-B244^T^ is most closely related to *Erwinia oleae* (DSM 23398^T^).

The highest 16S rRNA gene identity of SCU-B244^T^ with species of genera *Erwinia* and *Pantoea* were 95.43% (*Erwinia psidii* LMG 7039^T^) and 95.42% (*Pantoea calida* 1400/07^T^) respectively, phylogenic closest strain was *Erwinia oleae* (DSM 23398^T^) and the identity was 94.71%, which is lower than the 97% threshold that has been established to discriminate species. Strains showing less than 97% 16S rRNA gene identity are unlikely to have more than 60 to 70% DNA—DNA relatedness [23], and this level of rRNA sequence identity strongly suggests that SCU-B244^T^ is a novel species. The DNA—DNA relatedness between SCU-B244^T^ and *Erwinia oleae* (DSM 23398^T^) was 5.79 ± 2.52%. The value is mean of six hybridizations ± SD, which is significantly lower than the 70% value considered to be the threshold for the delineation of bacterial species [24]. ANI values between SCU-B244^T^ and related species are listed in Table 1. The values range from 72.42% to 74.41% which are lower than 95–96% ANI.

**Table 1:** OrthoANI values between SCU-B244^T^ and related species.

	1	2	3	4	5	6	7	8	9*	10*	11*	12*	13*	14*	15	16	17	18	19
1	100																		
2	73.82	100																	
3	73.62	77.41	100																
4	72.74	76.02	78.14	100															
5	73.72	90.53	77.72	76.30	100														
6	73.62	84.78	77.75	76.25	85.86	100													
7	74.03	77.14	78.05	76.23	77.45	76.86	100												
8	72.42	75.29	76.89	79.16	75.51	75.29	75.20	100											
9	74.07	76.72	78.62	77.06	76.98	76.91	76.94	76.09	100										
10	73.40	85.32	77.21	75.99	86.52	89.28	76.74	75.24	76.52	100									
11	73.75	77.26	83.21	77.94	77.46	77.55	77.56	76.94	78.91	77.10	100								
12	73.68	75.33	75.88	74.85	75.72	75.43	76.48	74.11	75.79	75.14	75.84	100							
13	72.68	74.69	74.98	74.09	74.99	74.66	75.88	73.54	74.62	74.46	74.75	77.58	100						
14	73.11	75.05	75.71	74.26	75.53	74.96	76.40	73.76	75.31	74.75	75.34	78.43	85.98	100					
15	73.23	75.20	76.05	74.62	75.28	75.17	76.25	73.92	76.14	74.83	75.37	87.99	77.63	78.29	100				
16	72.79	74.68	74.83	74.18	74.75	74.44	75.02	73.68	74.73	74.27	74.62	79.31	76.60	77.34	79.08	100			
17	74.41	76.12	76.23	75.36	76.42	75.93	77.05	74.34	76.32	75.84	76.41	79.37	82.36	83.36	79.05	77.80	100		
18	73.11	74.26	75.05	74.16	74.65	74.48	75.38	74.45	74.90	74.41	75.00	79.29	76.92	77.32	78.94	84.08	77.85	100	
19	73.43	75.34	75.74	74.75	75.72	75.50	76.44	74.08	75.69	75.02	75.65	87.77	77.76	78.61	90.67	79.14	79.23	79.31	100

Taxa: 1, SCU-B244^T^; 2, *Erwinia amylovora* NBRC 12687^T^; 3, *Erwinia billingiae* Eb661; 4, *Erwinia mallotivora* BT-MARDI; 5, *Erwinia pyrifoliae* DSM 12163^T^; 6, *Erwinia tasmaniensis* Et1 99^T^; 7, *Erwinia toletana* DAPP-PG 735; 8, *Erwinia tracheiphila* PSU-1; 9, *Erwinia oleae* DAPP-PG531^T^; 10, *Erwinia piriflorinigrans* CFBP 5888^T^; 11, *Erwinia typographi* M043b; 12, *Pantoea anthophila* 11–2; 13, *Pantoea rodasii* ND03; 14, *Pantoea rwandensis* ND04; 15, *Pantoea agglomerans* DAPP-PG734; 16, *Pantoea ananatis* LMG 2665^T^; 17, *Pantoea dispersa* EGD-AAK13; 18, *Pantoea stewartii* LMG 2715^T^; 19, *Pantoea vagans* C9-1

* Data from NCBI Genome, other data except 1 were taken from EzBioCloud

The DNA G+C content of strain SCU-B244^T^ was 55.32 mol% which could be discriminated from *Erwinia oleae* (DSM 23398^T^), which has a G+C content of 54.7 to 54.9 mol%.

API 20E and API 50CHE (BioMérieux) tests were also carried out and the results were compared to related strains (Table 2). The results revealed that SCU-B244^T^ strain can be discriminated from each recognized species of the genus *Erwinia* by at least three characteristics, and from the phylogenetically most closely related species, *Erwinia oleae*, by 10 API 50CHE characteristics (Table 3).

**Table 2:** Comparison of phenotypic characteristics between strain SCU-B244^T^, *Erwinia oleae* (DSM 23398^T^), and other related species.

Characteristic	1	2	3*	4*	5*	6*	7*	8*	9*	10^#^	11*	12*	13*	14*	15*	16^##^
Nitrate reduction	+	+	-	+	+	-	-	+	-	ND	-	+	-	-	-	ND
Fermentation of (API 50-CHE):																
Aesculin	+	+	-	+	+	-	+	+	+	-	-	+	-	+	-	+
L-Arabinose	+	+	+	+	+	-	+	+	+	+	+	+	+	+	+	+
D-Arabitol	-	+	-	-	+	-	-	-	-	-	-	-	-	+	+	-
Potassium Gluconate	+	+	-	+	-	-	+	-	-	+	-	-	-	-	-	+
Glycerol	+	-	-	+	+	-	-	+	+	+	+	+	+	+	+	+
Inositol	-	-	+	+	+	-	-	+	-	+	+	+	+	+	-	+
Potassium 2-Ketogluconate	+	+	-	+	-	-	-	-	-	-	-	-	-	-	-	+
D-Mannose	+	+	-	+	+	+	+	+	+	-	-	+	-	+	+	+
L-Rhamnose	+	+	-	+	+	-	-	+	+	-	-	+	-	-	-	+
D-Sorbitol	+	-	+	-	+	-	-	+	-	-	+	-	-	-	-	+
Sucrose	-	-	+	+	-	+	+	+	+	+	+	+	+	-	+	+
Xylitol	-	-	-	+	-	-	-	-	-	-	-	+	+	-	-	-

1, *Erwinia teleogrylli* sp. nov. SCU-B244^T^; 2, *E*. *oleae* DSM 23398^T^; 3, *E*. *amylovora* LMG 2024^T^; 4, *E*. *aphidicola* LMG 24877^T^; 5, *E*. *billingiae* LMG 2613^T^; 6, *E*. *mallotivora* LMG 2708^T^; 7, *E*. *papayae* CFBP 5189^T^; 8, *E*. *persicina* LMG 11254^T^; 9, *E*. *psidii* LMG 7039^T^; 10, *E*. *piriflorinigrans* CECT 7348^T^; 11, *E*. *pyrifoliae* ICMP 14143^T^; 12, *E*. *rhapontici* LMG 2688^T^; 13, *E*. *tasmaniensis* LMG 25318^T^; 14, *E*. *toletana* CFBP 6631^T^; 15, *E*. *tracheiphila* LMG 2707^T^; 16, *Pantoea gaviniae* LMG 26250^T^.

+, positive; -, negative; ND, not determined.

^#^ Data from López *et al*. [25].

* Data from Moretti *et al*. [22].

^##^ Data from Alexandra *et al*. [26]

**Table 3:** Differential phenotypic characteristics between SCU-B244^T^ and its closest phylogenetic neighbour, *Erwinia oleae* DSM 23398^T^.

Characteristic	SCU-B244^T^	*Erwinia oleae* DSM 23398^T^
**API 20E:**		
β-galactosidase activity	-	+
Citrate utilization	+	-
**API 50CHE:**		
Glycerol	+	-
D-Xylose	+	-
L-Sorbose	+	-
D-Sorbitol	+	-
Methyl-α-D-glucopyranoside	+	-
D-Cellobiose	+	-
D-Maltose	+	-
D-Melibiose	+	-
Gentiobiose	+	-
D-Arabitol	-	+
Potassium 5-ketogluconate	+	-

+, Positive; -, negative.

API ZYM enzymatic characteristics and BIOLOG GN2 carbon-source utilization results are presented as supplementary data (S1 and S2 Tables).

Fatty acid composition analysis of SCU-B244^T^ and related species of genera *Erwinia* and *Pantoea* is shown in Table 4. Strain SCU-B244^T^ contained C_16:0_ (28.2%), Summed feature 4 (12.4%), C_11:0_ 3-OH (11.6%), Summed feature 7 (10.5%), Summed feature 3 (10.0%), C_14:0_ (6.4%) as the major fatty acids, while strain DSM 23398^T^ contained C_16:0_ (38.8%), Summed feature 4 (32.7%), Summed feature 7 (9.1%), C_12:0_ (7.7%), C_16:0_ Δ^9^ cyclo (4.6%) as the major fatty acids (S3 Table). Summed feature 3 (10.0%), C_14:0_ 2-OH (3.7%) and C_18:0_ (3.7%) were detected from strain SCU-B244^T^ while those were not detected from strain DSM 23398^T^. Fatty acid composition of SCU-B244^T^ and reference strains data from *Erwinia* and *Pantoea* showing that most of the compositions were in the range of *Erwinia* and *Pantoea* [27], which indicates that SCU-B244^T^ belongs to group *Erwinia* and *Pantoea*.

**Table 4:** Fatty acid composition of *Erwinia teleogrylli* SCU-B244^T^ and reference strains from *Erwinia* and *Pantoea*. This table lists fatty acids including all fatty acid detected at a level higher than 0.5%. Summed feature 3 contained one or more of the following fatty acids: *iso*-C_16:1_, C_14:0_ 3-OH, C_12:0_ aldehyde and an unknown fatty acid ECL 10.928. Summed feature 4 contained one or more of the following fatty acids: C_16:1_ Δ^9^ cis and *iso*-C_15:0_ 2-OH. Summed feature 7 contained one or more of the following fatty acids: C_18:1_Δ^11^ cis, C_18:1_Δ^9^ trans and C_18:1_ Δ^6^ trans [27].

	Fatty acid composition (%)		
	*Erwinia teleogrylli*	Other *Erwinia* sp.*	*Pantoea* sp.*
	SCU-B244^T^	Range	Range
C_8:0_ 2-CH_2_CH_3_	0.6	ND	ND
C_11:0_ 3-OH	11.7	ND	ND
C_12:0_	4.3	3.2–5.9	3.3–4.4
C_14:0_	6.4	TR-5.8	TR-5.8
C_14:0_ 2-OH	3.7	ND	ND
C_14:1_ Δ^11^	1.1	ND	ND
C_15:0_	0.5	ND-TR	ND-1.6
C_16:0_	28.2	26.9–33.4	27.3–31.3
C_17:0_	ND	ND-1.2	ND-2.7
C_17:0_ cyclo	4.9	ND-13.6	ND-13.2
*iso*-C_18:0_	3.7	ND	ND
C_18:0_ 14-methyl	1.9	ND	ND
Summed feature 3*	10.0	ND-12.2	8.5–10.5
Summed feature 4*	12.4	22.7–33.7	10.8–24.4
Summed feature 7*	10.5	6.3–16.9	12.6–36.9

ND: Not Detected, TR: Trace amount (< 1.0%).

*Data from Mergaert *et al*. [27]

Based on the genotypic data (16S rRNA gene sequence analysis and MLSA), it is clear that SCU-B244^T^ is a member of genus *Erwinia*. DNA-DNA hybridization results, ANI values and phenotypic data obtained in this study can separate this isolate from related species. In conclusion the bacterial strain isolated from a cricket (genus *Teleogryllus*) represents a novel species for which the name *Erwinia teleogrylli* sp. nov. is proposed. Strain SCU-B244^T^ (= CGMCC 1.12772^T^ = DSM 28222^T^ = KCTC 42022^T^) is the type strain.

## Description of *Erwinia teleogrylli* sp. nov.

*Erwinia teleogrylli* (teleogryl'li. N.L. gen. n. *teleogrylli* of *Teleogryllus*, the insect from which the species was isolated).

The colonies are milky white, circular and convex with entire margins. Cells are short Gram-negative, facultatively anaerobic rods that are oxidase-negative, catalase-positive and able to grow in 6% (w/v) NaCl. Growth pH range is 6 to 9, with optimum growth at 7. Nitrate is reduced to nitrite.

Results obtained with API 20E tests (BioMérieux) gave positive results for acid production from citrate, glucose, mannitol, rhamnose, melibiose, amygdalin and arabinose, and negative results for β-galactosidase, gelatinase, urease, arginine digydrolase, lysine decarboxylase and ornithine decarboxylase activity, H_2_S and indole production, acid production from sorbitol and sucrose. According to API 50CHE tests (BioMérieux), acid is formed from glycerol, L-arabinose, D-ribose, D-xylose, D-galactose, D-glucose, D-fructose, D-mannose, L-sorbose, L-rhamnose, D-mannitol, D-sorbitol, methyl-αD-glucopyranoside, N-acetylglucosamine, arbutin, esculin ferric citrate, salicin, D-cellobiose, D-maltose, D-melibiose, D-trehalose, gentiobiose, potassium gluconate, potassium 2-ketogluconate, potassium 5-ketogluconate. No acid is produced from erythritol, D-arabinose, L-xylose, D-ardonitol, methyl-β D-xylopyranoside, dulcitol, inositol, methyl-αD-mannopyranoside, amygdalin, D-lactose, D-saccharose, inulin, D-melezitose, D-raffinose, amidon, glycogen, xylitol, D-turanose, D-lyxose, D-tagatose, D-fucose, L-fucose, D-arabitol, L-arabitol. Results obtained with API ZYM tests (BioMérieux) gave positive enzymatic activity results for alkaline phosphatase, esterase (C4), esterase lipase (C8), acid phosphatase, naphthol-AS-B1-phosphohydrolase, β-galactosidase, β- glucosidase and N-acetyl-β- glucosaminidase, and negative results for lipase (C14), cystine arylamidase, trypsin, α-chymotrypsin, β-glucuronidase, α-glucosidase, α-mannosidase and α-fucosidase.

The following substrates were utilized according to BIOLOG GN2 carbon-source utilization analysis: dextrin, N-acetyl-D-glucosamine, L-arabinose, D-fructose, D-galactose, α-D-glucose, maltose, D-mannitol, D-mannose, D-melibiose, β-methyl-D-glucoside, D-psicose, L-rhamnose, D-trehalose, turanose, pyruvic acid methyl ester, citric acid, formic acid, D-galactonic acid lactone, D-galacturonic acid, D-gluconic acid, p-hydroxy phenylacetic acid, α-keto glutaric acid, propionic acid, D-saccharic acid, L-alanine, L-alanyl-glycine, L-asparagine, L-aspartic acid, L-glutamic acid, glycyl-L-aspartic acid, glycyl-L-glutamic acid, L-proline, L-serine, inosine, uridine, thymidine, glycerol, D,L-α-glycerol phosphate, glucose-1-phosphate, glucose-6-phosphate.

Major fatty acids are C_16:0_ (28.2%), Summed feature 4 (12.4%), C_11:0_ 3-OH (11.6%), Summed feature 7 (10.5%) and Summed feature 3 (10.0%), with other minor components, including C_14:0_ (6.4%), C_17:0_ Δ^9^ cyclo (4.9%), C_12:0_ (4.3%), C_14:0_ 2-OH (3.7%), iso-C_18:0_ (3.6%), C_18:0_ 14-methyl (1.9%), C_14:1_ Δ^11^ (1.1%), C_8:0_ 2-CH_2_CH_3_ (0.6%), C_15:0_ (0.5%). The DNA G+C content is 55.32 mol%.

The type strain, SCU-B244^T^ (= CGMCC 1.12772^T^ = DSM 28222^T^ = KCTC 42022^T^), was isolated from crickets (genus *Teleogryllus*) sampled in Chengdu (30°33’ N, 103°58’ E, altitude 495m), Sichuan Province, China.

## Supporting Information

S1 Certification Deposit certification of KCTC. (PDF)

S2 Certification Deposit certification of CGMCC. (PDF)

S1 Fig Neighbour-joining tree based on almost complete 16S rRNA gene sequences related strains. The diagram shows the phylogenetic relationship between *Erwinia teleogrylli* sp. nov. and the first 66 hit strains at the EzTaxon server within the family *Enterobacteriaceae*. Bar, 0.5% nucleotide substitutions. Numbers at branching points are bootstrap percentage values based on 1000 replications. Only values >50% are shown.
(TIF)

S2 Fig Maximum-likelihood tree based on almost complete 16S rRNA gene sequences related strains. The diagram shows the phylogenetic relationship between *Erwinia teleogrylli* sp. nov. and the first 66 hit strains at the EzTaxon server within the family *Enterobacteriaceae*. Bar, 0.5% nucleotide substitutions. Numbers at branching points are bootstrap percentage values based on 1000 replications. Only values >50% are shown.
(TIF)

S3 Fig Neighbour-joining tree based on almost complete 16S rRNA gene sequences of the genera *Erwinia* and *Pantoea*. The diagram shows the phylogenetic relationship between *Erwinia teleogrylli* sp. nov. and taxa related type species of the genera *Erwinia* and *Pantoea* except *Candidatus* Erwinia dacicola. *Escherichia coli* ATCC 11775^T^ was used as the outgroup. Bar, 0.5% nucleotide substitutions. Numbers at branching points are bootstrap percentage values based on 1000 replications. Only values >50% are shown.
(TIF)

S4 Fig Neighbour-joining tree based on concatenated partial *atpD*, *gyrB* and *infB* sequences of the genera Erwinia, Pantoea and Tatumella. The diagram shows phylogenetic relationship between *Erwinia teleogrylli* sp. nov. and taxa related species of genera *Erwinia*, *Pantoea* and *Tatumella*. Bar, 0.5% nucleotide substitutions. Numbers at branching points are bootstrap percentage values based on 1000 replications. Only values >50% are shown.
(TIF)

S1 Table API ZYM enzymatic characteristics of strain SCU-B244^T^. (DOCX)

S2 Table BIOLOG GN2 carbon-source utilization analysis results of strain SCU-B244^T^. (DOCX)

S3 Table Fatty acid compositions comparison of the strain SCU-B244^T^ and DSM 23398^T^. (DOCX)
