# Supplementary material for: Erwinia teleogrylli sp. nov., a Bacterial Isolate Associated with a Chinese Cricket
Source: PLoS One. 2016 Jan 22;11(1):e0146596. doi: 10.1371/journal.pone.0146596 (PMC4723187; doi:10.1371/journal.pone.0146596)
Supplement: S1 Certification — (PDF) [file pone.0146596.s001.pdf]

Korean Collection for Type Cultures (KCTC), Microbial Resource Center
Korea Research Institute of Bioscience and Biotechnology (KRIBB)
125 Gwahak-ro, Yuseong-gu, Daejeon, 305-806, Korea
Tel: +82-42-860-4629, FAX: +82-42-860-4625

Certificate of Deposit

Date: February 11, 2014

The following information is confidential and acts only to confirm that a strain has been deposited to the KCTC.

***Dickeya* sp. SCU-B244** has been deposited in the general collection of microorganisms in the KCTC under the number **KCTC 42022**.

This strain is now readily available to the international scientific research community. Access to the strain is not restricted and it shall be supplied to anyone upon request.

Song-Gun Kim

Song-Gun Kim, Ph.D.
Curator responsible for the strain
Korean Collection for Type Cultures (KCTC)
Microbial Resource Center (BRC)
Korea Research Institute of Bioscience and Biotechnology (KRIBB)
125 Gwahak-ro, Yuseong-gu, Daejeon, 305-806, Korea
