# Supplementary material for: Erwinia teleogrylli sp. nov., a Bacterial Isolate Associated with a Chinese Cricket
Source: PLoS One. 2016 Jan 22;11(1):e0146596. doi: 10.1371/journal.pone.0146596 (PMC4723187; doi:10.1371/journal.pone.0146596)
Supplement: S2 Certification — (PDF) [file pone.0146596.s002.pdf]

中国微生物菌种保藏管理委员会普通微生物中心
China General Microbiological Culture Collection Center (CGMCC)

Address: Institute of Microbiology, Chinese Academy of Sciences, Datun Road, Chaoyang District, Beijing 100101, China
Telephone: 86-10-64807355 Fax: 86-10-64807288 E-mail: cgmcc@sun.im.ac.cn Homepage: www.cgmcc.net

受理通知书

NOTIFICATION OF RECEIPT

CGMCC No. 1.12772

1. Name and address of the depositor or agent

刘波 Bo LIU
College of Light Industry, Textile and Food Engineering, Sichuan University
No. 24 Southern Yihuan, Chengdu 610065, P.R. China

2. Strain reference given by depositor

SCU-B244

3. Deposited microorganisms appended

☐ Scientific description

■ Proposed taxonomic name

Dickeya sp.

4. The deposited microorganism has been received and numbered as CGMCC No. 1.12772 on January, 2014. The strain has been checked for viability in the CGMCC and is stored using one of the standard methods used in the CGMCC.

5. This strain is available in the public accessible section of the CGMCC and restrictions have not been placed on access. It will be included in the published and online catalogue after publication of this number by the authors.

Signature of Head of CGMCC Yu-Guang ZHOU

中国微生物菌种保藏管理委员会普通微生物中心

Date February 19, 2014
