# Supplementary figures and images for: Erwinia teleogrylli sp. nov., a Bacterial Isolate Associated with a Chinese Cricket
Source: PLoS One. 2016 Jan 22;11(1):e0146596. doi: 10.1371/journal.pone.0146596 (PMC4723187; doi:10.1371/journal.pone.0146596)

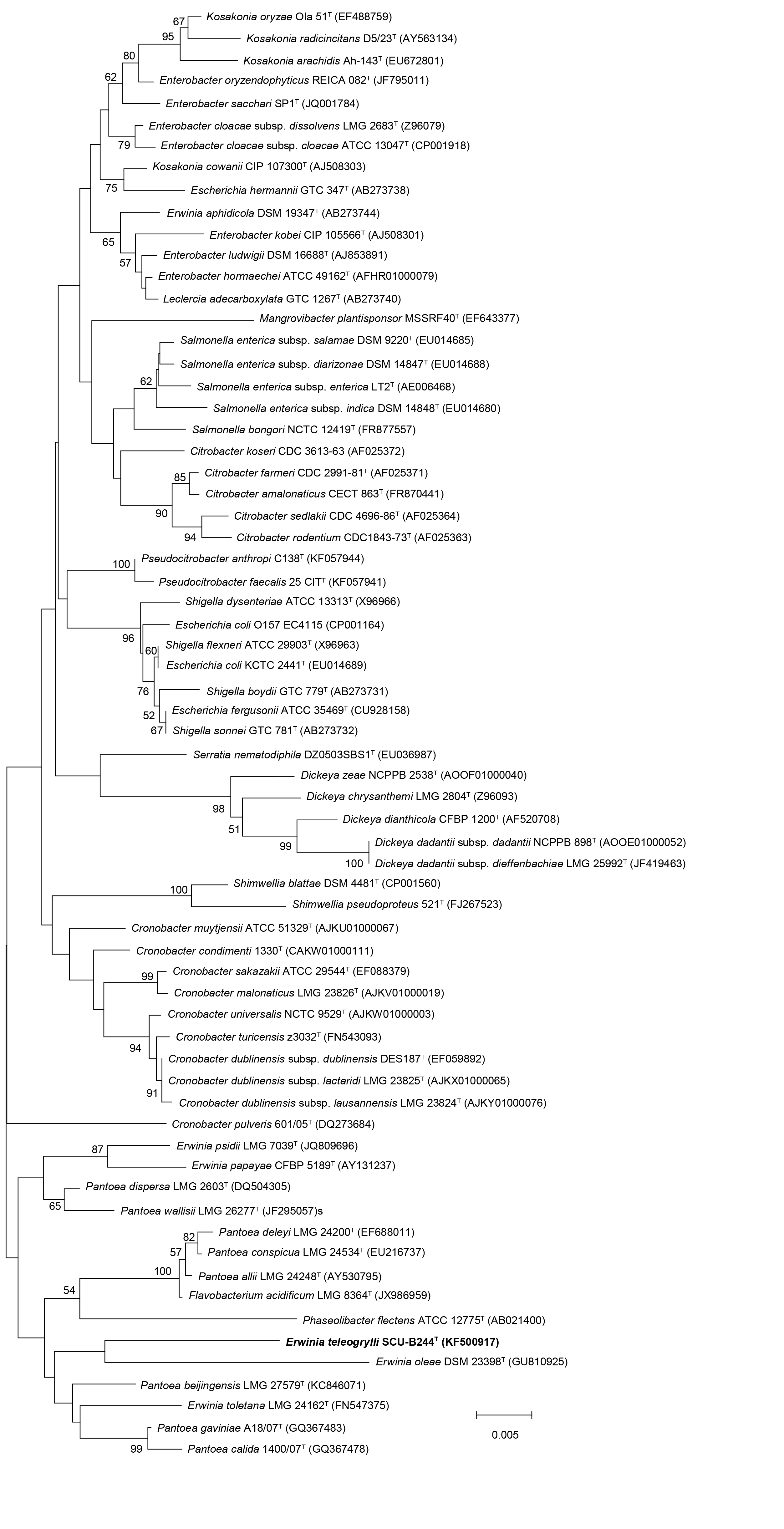

Supplement: S1 Fig — The diagram shows the phylogenetic relationship between Erwinia teleogrylli sp. nov. and the first 66 hit strains at the EzTaxon server within the family Enterobacteriaceae. Bar, 0.5% nucleotide substitutions. Numbers at branching points are bootstrap percentage values based on 1000 replications. Only values >50% are shown. (TIF) [file pone.0146596.s003.tif]

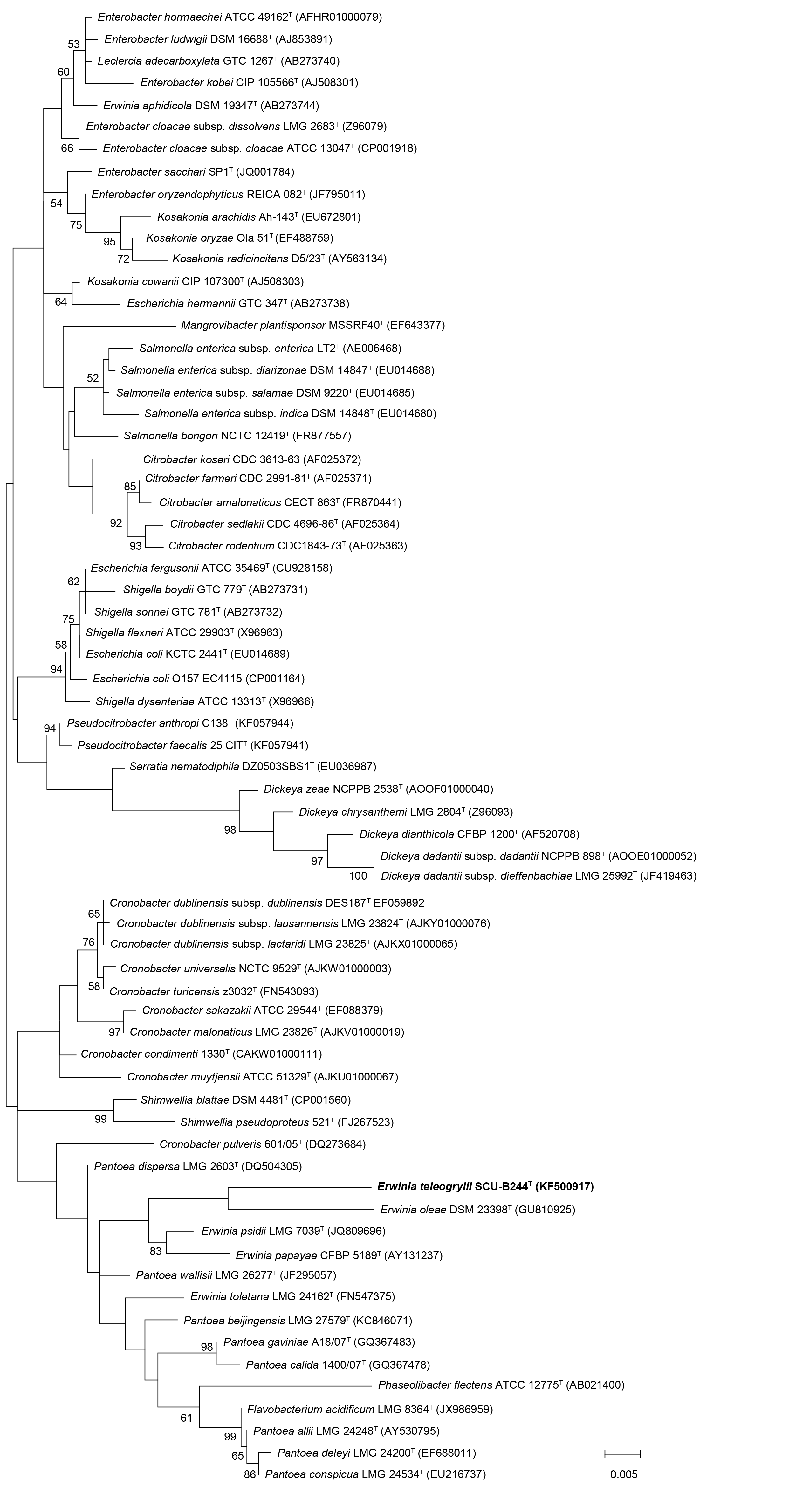

Supplement: S2 Fig — The diagram shows the phylogenetic relationship between Erwinia teleogrylli sp. nov. and the first 66 hit strains at the EzTaxon server within the family Enterobacteriaceae. Bar, 0.5% nucleotide substitutions. Numbers at branching points are bootstrap percentage values based on 1000 replications. Only values >50% are shown. (TIF) [file pone.0146596.s004.tif]

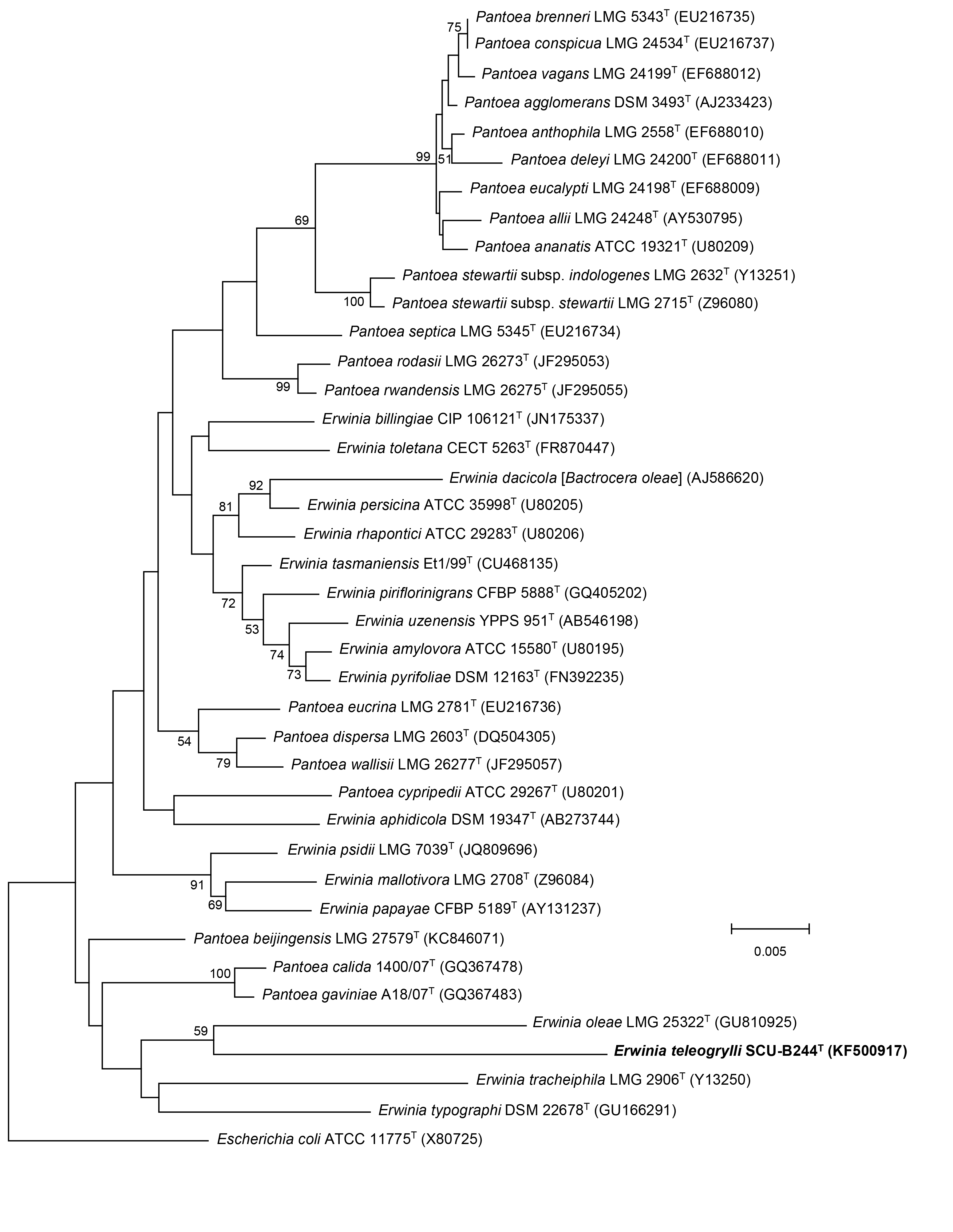

Supplement: S3 Fig — The diagram shows the phylogenetic relationship between Erwinia teleogrylli sp. nov. and taxa related type species of the genera Erwinia and Pantoea except Candidatus Erwinia dacicola. Escherichia coli ATCC 11775T was used as the outgroup. Bar, 0.5% nucleotide substitutions. Numbers at branching points are bootstrap percentage values based on 1000 replications. Only values >50% are shown. (TIF) [file pone.0146596.s005.tif]

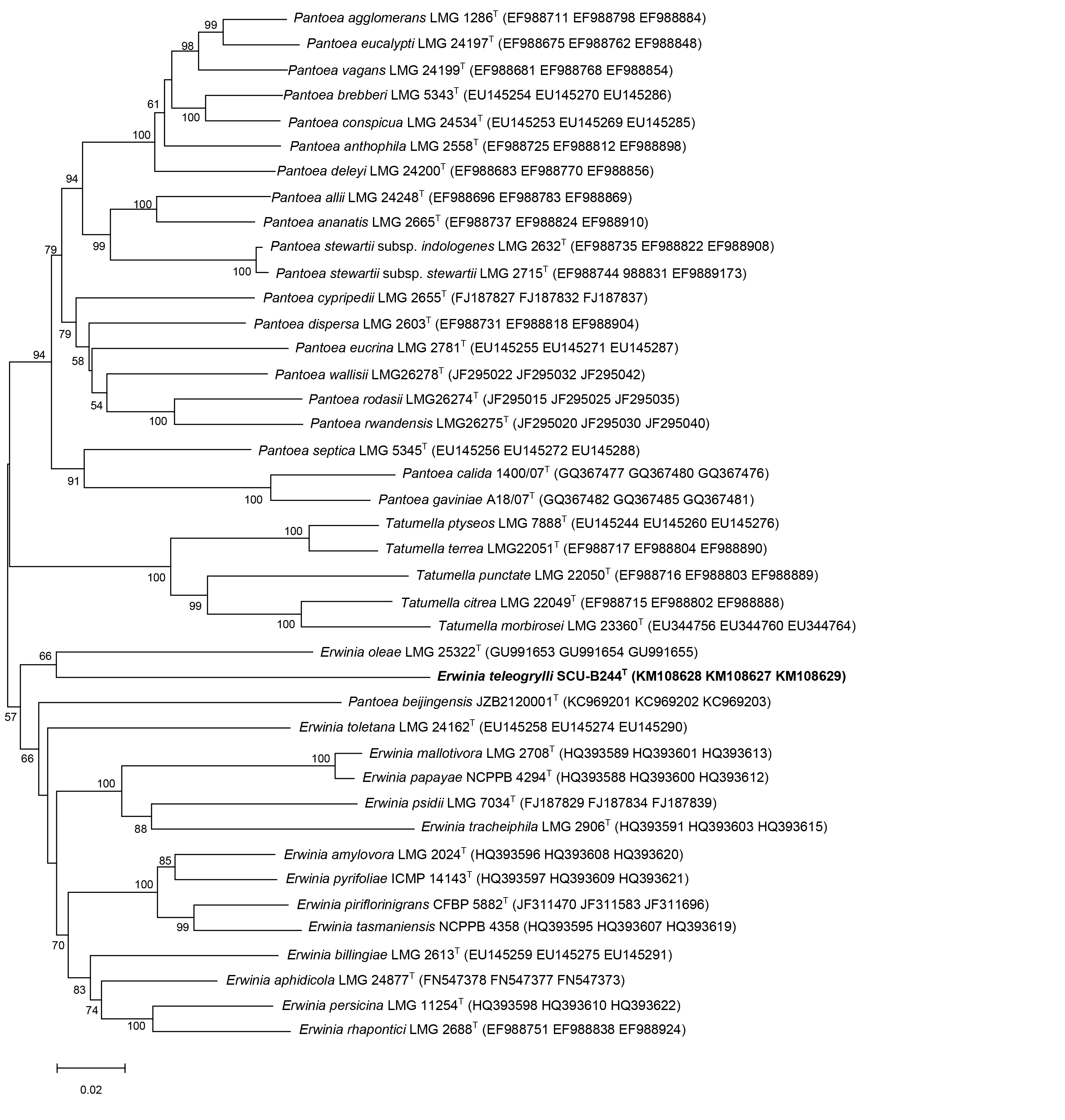

Supplement: S4 Fig — The diagram shows phylogenetic relationship between Erwinia teleogrylli sp. nov. and taxa related species of genera Erwinia, Pantoea and Tatumella. Bar, 0.5% nucleotide substitutions. Numbers at branching points are bootstrap percentage values based on 1000 replications. Only values >50% are shown. (TIF) [file pone.0146596.s006.tif]
