# Supplementary material for: Erwinia teleogrylli sp. nov., a Bacterial Isolate Associated with a Chinese Cricket
Source: PLoS One. 2016 Jan 22;11(1):e0146596. doi: 10.1371/journal.pone.0146596 (PMC4723187; doi:10.1371/journal.pone.0146596)
Supplement: S1 Table — (DOCX) [file pone.0146596.s007.docx]

**S1 Table API ZYM enzymatic characteristics test for strain SCU-B244^T^.**

| API ZYM | SCU-B244^T^ |
| --- | --- |
| Alkaline phosphatase | + |
| Esterase(C4) | + |
| Esterase lipase(C8) | + |
| Lipase (C14) | - |
| Leucine arylamidase | ± |
| Valine arylamidase | ± |
| Cystine arylamidase | - |
| Trypsin | - |
| α-chymotrypsin | - |
| Acid phosphatase | + |
| Naphthol-AS-B1-phosphohydrolase | + |
| α-galactosidase | ± |
| β-galactosidase | + |
| β- glucuronidase | - |
| α-glucosidase | - |
| β- glucosidase | + |
| N-acetyl-β- glucosaminidase | + |
| α-mannosidase | - |
| α-fucosidase | - |

+, positive; -, negative; ±, variational result.
