# Supplementary material for: Erwinia teleogrylli sp. nov., a Bacterial Isolate Associated with a Chinese Cricket
Source: PLoS One. 2016 Jan 22;11(1):e0146596. doi: 10.1371/journal.pone.0146596 (PMC4723187; doi:10.1371/journal.pone.0146596)
Supplement: S2 Table — (DOCX) [file pone.0146596.s008.docx]

**S2 Table BIOLOG GN2 carbon-source utilization analysis results for strain SCU-B244^T^.**

| Carbon-source | SCU-B244^T^ | Carbon-source | SCU-B244^T^ |
| --- | --- | --- | --- |
| Water | - | p-Hydroxy Phenylacetic Acid | + |
| α-Cyclodextrin | - | Itaconic Acid | - |
| Dextrin | + | α-Keto Butyric Acid | - |
| Glycogen | * | α-Keto Glutaric Acid | + |
| Tween 40 | - | α-Keto Valeric Acid | - |
| Tween 80 | - | D,L-Lactic Acid | - |
| N-Acetyl-D-Galactosamine | - | Malonic Acid | - |
| N-Acetyl-D-Glucosamine | + | Propionic Acid | + |
| Adonitol | - | Quinic Acid | - |
| L-Arabinose | + | D-Saccharic Acid | + |
| D-Arabitol | - | Sebacic Acid | - |
| D-Cellobiose | - | Succinic Acid | * |
| i-Erythritol | - | Bromo Succinic Acid | * |
| D-Fructose | + | Succinamic　Acid | - |
| L-Fucose | - | Glucuronamide | * |
| D-Galactose | + | L-Alaninamide | - |
| Gentiobiose | - | D-Alanine | - |
| α-D-Glucose | + | L-Alanine | + |
| m-Inositol | - | L-Alanyl-glycine | + |
| α-D-Lactose | - | L-Asparagine | + |
| Lactulose | - | L-Aspartic Acid | + |
| Maltose | + | L-Glutamic　Acid | + |
| D-Mannitol | + | Glycyl-L-AsparticAcid | + |
| D-Mannose | + | Glycyl-L-Glutamic Acid | + |
| D-Melibiose | + | L-Histidine | - |
| β-Methyl-D-Glucoside | + | Hydroxy-L-Proline | - |
| D-Psicose | + | L-Leucine | - |
| D-Raffinose | - | L-Ornithine | - |
| L-Rhamnose | + | L-Phenylalanine | - |
| D-Sorbitol | - | L-proline | + |
| Sucrose | - | L-Pyroglutamic Acid | - |
| D-Trehalose | + | D-Serine | - |
| Turanose | + | L-Serine | + |
| Xylitol | - | L-Threonine | * |
| Pyruvic Acid Methyl Ester | + | D,L-Carnitine | - |
| Succinic Acid Mono-Methyl Ester | - | γ-Amino Butyric Acid | - |
| Acetic Acid | * | Urocanic Acid | - |
| Cis-Aconitic Acid | - | Inosine | + |
| Citric Acid | + | Uridine | + |
| Formic Acid | + | Thymidine | + |
| D-Galactonic Acid Lactone | + | Phenyethylamine | - |
| D-Galacturonic Acid | + | Putrescine | - |
| D-Gluconic Acid | + | 2-Aminoethanol | - |
| D-Glucosaminic Acid | - | 2,3-Butanediol | - |
| D-Glucuronic Acid | - | Glycerol | + |
| α-Hydroxy Butyric Acid | - | D,L-α-glycerol Phosphate | + |
| β-Hydroxy Butyric Acid | - | Glucose-1-Phosphate | + |
| γ-Hydroxy Butyric Acid | - | Glucose-6-Phosphate | + |

+, positive; -, negative; *, not determined
