# Supplementary material for: Erwinia teleogrylli sp. nov., a Bacterial Isolate Associated with a Chinese Cricket
Source: PLoS One. 2016 Jan 22;11(1):e0146596. doi: 10.1371/journal.pone.0146596 (PMC4723187; doi:10.1371/journal.pone.0146596)
Supplement: S3 Table — (DOCX) [file pone.0146596.s009.docx]

**S3 Table Fatty acid compositions comparison of the strain SCU-B244^T^ and DSM 23398^T^.**

| Fatty acids | SCU-B244^T^ | DSM 23398^T^ |
| --- | --- | --- |
| C_16:0_ | 28.20% | 38.79% |
| C_16:1_ Δ^9^ | 12.44% | 32.69% |
| C_11:0_ 3-OH | 11.67% | 3.99% |
| C_18:1_ Δ^9^ | 10.54% | 9.13% |
| C_14:0_ 3-OH | 10.01% | ND |
| C_14:0_ | 6.43% | 0.63% |
| C_17:0_ Δ^9^ cyclo | 4.94% | ND |
| C_12:0_ | 4.33% | 7.68% |
| C_14:0_ 2-OH | 3.70% | ND |
| *iso*-C_18:0_ | 3.67% | ND |
| C_18:0_ 14-methyl | 1.90% | ND |
| C_14:1_ Δ^11^ | 1.09% | 0.55% |
| C_8:0_ 2-CH_2_CH_3_ | 0.58% | ND |
| C_15:0_ | 0.50% | 0.27% |
| C_16:0_ Δ^9^ cyclo | ND | 4.61% |
| C_18:0_ | ND | 1.33 |
| C_17:0_ | ND | 0.33 |

ND: Not Detected
